# HSP90 Co-Chaperone, CacyBP/SIP, Protects α-Synuclein from Aggregation

**DOI:** 10.3390/cells9102254

**Published:** 2020-10-08

**Authors:** Anastasiia Bohush, Anna Filipek

**Affiliations:** Nencki Institute of Experimental Biology, Polish Academy of Sciences, 3 Pasteur Street, 02-093 Warsaw, Poland; a.bohush@nencki.edu.pl

**Keywords:** α-synuclein, CacyBP/SIP, protein aggregation, Parkinson’s disease

## Abstract

Recently, it has been found that the CacyBP/SIP protein acts as HSP90 co-chaperone and exhibits chaperone properties itself. Namely, CacyBP/SIP has been shown to protect citrate synthase from aggregation and to recover the activity of thermally denatured luciferase in vitro. In the present work, we have analyzed the influence of CacyBP/SIP on aggregation of α-synuclein, a protein present in Lewy bodies of Parkinson’s disease brain. By applying a thioflavin T (ThT) fluorescence assay, we have found that CacyBP/SIP protects α-synuclein from aggregation and that the fragment overlapping the N-terminal part and the CS domain of CacyBP/SIP is crucial for this activity. This protective effect of CacyBP/SIP has been confirmed by results obtained using high-speed ultracentrifugation followed by dot-blot and by transmission electron microscopy (TEM). Interestingly, CacyBP/SIP exhibits the protective effect only at the initial phase of α-synuclein aggregation. In addition, we have found that, in HEK293 cells overexpressing CacyBP/SIP, there are less α-synuclein inclusions than in control ones. Moreover, these cells are more viable when treated with rotenone, an agent that mimics PD pathology. By applying proximity ligation assay (PLA) on HEK293 cells and in vitro assays with the use of purified recombinant proteins, we have found that CacyBP/SIP directly interacts with α-synuclein. Altogether, in this work, we show for the first time that CacyBP/SIP is able to protect α-synuclein from aggregation in in vitro assays. Thus, our results point to an important role of CacyBP/SIP in the pathology of Parkinson’s disease and other synucleinopathies.

## 1. Introduction

α-synuclein, a protein encoded by the *SNCA* gene, is abundantly expressed in the mammalian brain. Numerous studies have shown that, under physiological conditions, α-synuclein is involved in storage and recycling of neurotransmitters, synaptic plasticity, mitochondrial function, and glucose metabolism [1,2,3,4]. Under pathology such as Parkinson’s disease (PD) α-synuclein forms aggregates, which are mainly present in Lewy bodies (LBs) in substantia nigra. In addition, mutations in the gene encoding α-synuclein are known to be linked to PD development.

Different methods, including NMR, have shown that α-synuclein is unstructured and thus it is classified to the group of intrinsically disordered proteins [5]. In the cell, α-synuclein may exist as the monomer, oligomer, and/or in the form of aggregates. Misfolded oligomeric α-synuclein is considered to be toxic for the cell [6]. It disrupts various signaling pathways and contributes to development of PD and other synucleinopathies [7]. For instance, through affecting the mitochondrial membrane, oligomers of α-synuclein lead to reactive oxygen species (ROS) generation and mitochondrial respiration damage [8]. They also alter mitochondrial architecture and activity of complex I. In addition, α-synuclein oligomers induce formation of ion channels in the plasma membrane, which, consequently, alters Ca^2+^ influx and compromises cellular homeostasis [9,10]. Numerous observations suggest that α-synuclein and some other misfolded proteins, such as tau, propagate, in a prion like manner, spreading the pathology throughout the brain [11,12].

So far, multiple tools aiming at reducing α-synuclein synthesis/accumulation (e.g., siRNA, immunotherapy) and preventing, or at least mitigating, α-synuclein aggregation have been elaborated in vitro and in animal models. In the latter respect, a number of small molecules that bind to different regions of α-synuclein and interfere with aggregation kinetics have been identified [13]. One of the cellular mechanisms that control α-synuclein folding and may prevent its pathological aggregation involves chaperone family proteins/heat shock proteins (HSPs) [14,15]. Several members of this family have been linked to α-synuclein aggregation both in vitro and in brains of PD patients. For example, it has been reported that the HSP90 chaperone inhibits formation of α-synuclein fibrils [16,17] and that the level of this chaperone changes in PD pathology [18]. In addition, it has been found that the activation of heat shock factor-1 (HSF-1), which regulates numerous HSP encoding genes, is abolished in aged cells [19].

The function/activity of HSP90 is regulated by many co-chaperones of which CacyBP/SIP has been identified only recently [20,21]. CacyBP/SIP is highly expressed in the brain, where it is mainly localized in neurons [22]. The protein is composed of three major domains. Two of them, the N-terminal and central CS (residues 1–77 and residues 74–178, respectively), have a globular nature while the C-terminal, also called the SGS domain (residues 178–229), is unstructured [23]. Recently, it has been found that CacyBP/SIP protects citrate synthase from aggregation and recovers the activity of thermally denatured luciferase in vitro [20]. Importantly, the recovery rate of luciferase activity in the presence of CacyBP/SIP alone was similar to that obtained for HSP90, which suggests that CacyBP/SIP itself exhibits chaperone activity. It has been also shown that CacyBP/SIP protects cells from different kinds of stress [21]. These in vitro results were substantiated by in vivo studies, which showed that the level of CacyBP/SIP was higher in some brain structures of stressed mice. This observation suggests an important role of this protein in cellular response to stress. Upregulation of CacyBP/SIP has been found in some neurodegenerative disorders such as frontotemporal dementia (FTD), amyotrophic lateral sclerosis (ALS) [24] or Huntington’s disease (HD) [25]. It is quite noteworthy that recent results of mass spectrometry-based quantitative proteomics have shown a decreased CacyBP/SIP expression in some brain areas of PD patients [26]. Taking into account that CacyBP/SIP is abundantly expressed in mammalian brain, in this work, we analyzed its influence on α-synuclein aggregation in in vitro assays, using purified recombinant proteins, and in HEK293 cells.

## 2. Materials and Methods

The presented research was performed in compliance with ethical standards.

### 2.1. Plasmids

Plasmids used in this study were described earlier (Table 1) except for pET28a-α-synuclein, which was prepared as follows: the fragment containing a coding sequence of human α-synuclein was amplified by PCR using pcDNA4-α-synuclein-3xFLAG plasmid (kindly provided by Dr. U. Dettmer, Harvard Medical School, Boston, USA) as a template and the following primers: forward-5′-GCAGCCATATGGATGTATTCATGAAAGGACTTTC-3′ and reverse- 5′-CCGCAAGCTTTTAGGCTTCAGGTTCGTAGTC-3′. The PCR product was digested with NdeI and HindIII restriction enzymes (both from Thermo Fisher Scientific, Waltham, MA, USA) and introduced into the pET28a plasmid (Sigma-Aldrich, St. Louis, MO, USA) previously digested with the same enzymes. After DNA sequencing (Institute of Biochemistry and Biophysics, PAS, Warsaw, Poland), the correctness of the sequence of the cloned insert was confirmed using the BLAST software.

### 2.2. Proteins

HSP90 was purchased from Enzo Life Sciences, Farmingdale, NY, USA. Other proteins were expressed in *E. coli* Rosetta strain (Novagen, Merck Millipore, Burlington, MA, USA). His-tagged CacyBP/SIP and its domains were expressed and purified as previously described [27]. α-synuclein was purified as follows. Bacteria were transformed with pET28a-α-synuclein plasmid and incubated overnight at 37 °C in LB media supplemented with kanamycin (Sigma-Aldrich). Then, the culture was scaled up and left to grow until OD_600_ reached 0.6. Synthesis of α-synuclein was induced with IPTG (Sigma-Aldrich) at 0.4 mM final concentration and bacteria were grown at 37 °C for 4 h with agitation. After that, bacteria were harvested by centrifugation at 6000 *g* at 4 °C and resuspended in the binding buffer (BB) containing 50 mM Tris, 1 mM EGTA, and 1 mM DTT, pH 7.5, supplemented with protease inhibitor cocktail (Sigma-Aldrich), according to the manufacturer’s protocol. Cells were sonicated (using an S-250D Branson Ultrasonic apparatus, Brookfield, CT, USA) for 3 min (15 s “ON” and 15 s “OFF”, at 30% of power) on ice. The homogenate was centrifuged at 35,000 rpm (Coulter’s Optima L-100XP ultracentrifuge, 70Ti Fixed-Angle Titanium Rotor, Beckman, Brea, CA, USA) for 1 h. The resulting supernatant was incubated for 10 min at 92 °C in a water bath [28], chilled on ice and centrifuged again as described above. The supernatant (heat stable fraction) was then applied onto the DEAE-Sepharose (Sigma-Aldrich) resin equilibrated with BB. The resin was washed with BB containing NaCl at 0, 0.05, 0.1 or 0.15 M concentration. Fraction enriched in α-synuclein, eluted with buffer containing 0.35 M NaCl, was then dialyzed overnight at 4 °C against buffer containing 20 mM Tris and 150 mM NaCl, pH 7.5. The concentration of α-synuclein was estimated using BCA Protein Assay Kit (Pierce, Thermo Fisher Scientific) and concentration of other proteins by Bradford’s procedure (Bio-Rad, Hercules, CA, USA).

### 2.3. In Vitro Aggregation ThT Assay and Dot-Blot Analysis

Aggregation of α-synuclein was performed as described previously [29]. 30 µM α-synuclein was mixed with 30 µM, 5 µM, 1 µM, or 0.5 µM CacyBP/SIP in fibrillation buffer (FB) containing 10 mM HEPES, 100 mM NaCl, 1 mM PMSF, 0.02% (*w*/*v*) NaN_3_, pH 7.5 and incubated at 37 °C in a thermomixer (ThermoMixer C, Eppendorf, Hamburg, Germany) with 400 rpm agitation for 11 days. NaN_3_ and PMSF were added to the fibrillation buffer to inhibit bacterial growth and activity of proteases, respectively. Every 24 h, a 150 µL aliquot of each sample was taken and premixed with thioflavin T (ThT) (Sigma-Aldrich) at 10 µM final concentration in a well of a 96-well black plate (Greiner Bio-One, Monroe, NC, USA). After incubation at RT for 15 min in the darkness, the ThT fluorescence (excitation 440 nm, emission 485 nm) was measured in a microplate reader (Tecan, Morrisville, NC, USA).

In order to perform dot-blot analysis, the mixture of α-synuclein with CacyBP/SIP (150 µL) on day 4 of incubation was taken and ultracentrifuged at 100,000 rpm for 1 h at 4 °C (Sorval MTX 150 Series Micro Ultracentrifuge, S120-AT3 Fixed Angle Rotor, Thermo Fisher Scientific). Then, the soluble fraction was diluted 1:1 in H_2_O and 5 µL of such mixture was applied on a nitrocellulose membrane (0.45 µm pore size, Bio-Rad). After drying (about 5 min), the membrane was blocked in 5% (*w*/*v*) milk in TBS-T at RT for 1 h. Primary mouse anti-α-synuclein antibody (Abcam, Cambridge, UK), diluted 1:3000, was added and incubation was carried out at RT for 2 h. Then, the membrane was washed 3 times for 5 min in TBS-T and secondaryanti-mouse antibody (Jackson ImmunoResearch, West Grove, PA, USA) conjugated with horseradish peroxidase (HRP), diluted 1:10,000, was added at RT for 1 h.

### 2.4. Transmission Electron Microscopy (TEM)

Samples of α-synuclein taken on day 4 of incubation were applied to 400-mesh cooper grids (TedPella, Redding, CA, USA). Subsequently, negative staining with 2% (*w*/*v*) aqueous solution of uranyl acetate (SPI Supplies, West Chester, PA, USA) was performed. The micrographs were collected by means of a high-performance transmission electron JEM 1400 microscope (JEOL Ltd., Akishima, Japan) equipped with 11 Megapixel TEM Camera MORADA G2 (EMSIS GmbH, Münster, Germany) in the Laboratory of Electron Microscopy at the Nencki Institute of Experimental Biology PAS, Warsaw, Poland.

### 2.5. Enzyme-Linked Immunosorbent Assay (ELISA)

3.5 μg of α-synuclein or BSA as negative control (Sigma-Aldrich) in 50 µL of coating buffer (100 mM Na_2_HPO_4_ and 100 mM NaH_2_PO_4_, pH 8.0) were immobilized in wells of a 96-well plate. After overnight incubation with gentle agitation at 4 °C, the solution was removed and wells were washed with PBS containing 0.05% Tween 20 (PBS-T). The remaining adsorption sites were blocked with PBS-T containing 10% BSA (Sigma-Aldrich) at RT for 3 h. After rinsing, the wells with washing buffer increasing amounts of purified recombinant CacyBP/SIP, in a stoichiometric ratio relative to α-synuclein, were added in reaction buffer (10 mM Tris, 1 mg/mL BSA, 5% glycerol, 10 mM NaCl, pH 8.0). After overnight incubation with gentle agitation at 4 °C, wells were washed as above and primary antibody against CacyBP/SIP (Cell Signaling Technology, Danvers, MA, USA), diluted 1:4000 in PBS-T, was added. After 3 h of incubation in the above conditions, wells were washed again and then secondary anti-rabbit antibody conjugated with horseradish peroxidase (HRP) (Merck Millipore), diluted 1:12,000 in PBS-T, was added. After 2 h of incubation, wells were washed again and the absorbance of a chromogenic HRP substrate (TMB peroxidase EIA substrate kit, Bio-Rad) was registered at 450 nm using a microplate reader (Tecan).

### 2.6. Crosslinking Experiment

α-synuclein (30 μM) was mixed with CacyBP/SIP (30 μM) in 15 μL of buffer containing 10 mM HEPES, 100 mM NaCl, 0.02% (*w*/*v*) NaN_3_, pH 7.5. The zero-length crosslinking reagent, EDC (2.5 mM final concentration), supplemented with NHS (5 mM final concentration) (both from Sigma-Aldrich), was added from fresh stock. In control reactions, CacyBP/SIP or α-synuclein was cross-linked alone or was incubated without the crosslinker. After 2 h incubation at RT, the reaction was terminated by addition of Laemmle’s sample buffer and the protein samples were analyzed by SDS-PAGE.

### 2.7. Cell Culture and Transfection

HEK293 cells (ATCC CRL-1573^TM^, Sigma-Aldrich) were cultured in Dulbecco’s Modified Eagle Medium (DMEM, Sigma-Aldrich) containing 10% (*w*/*v*) fetal bovine serum (FBS) (Gibco, Thermo Fisher Scientific), penicillin (100 U/mL) and streptomycin (100 µg/mL) (both from Sigma-Aldrich) at 37 °C and under 5% CO_2_. Morphology of cells was monitored every day under the light microscope (TMS, Nikon, Melville, NY, USA). Medium was exchanged every 2 days and, every 4 days, cells were passaged on a new plate. Cells (75–80% confluent) were transfected with an appropriate plasmid (Table 1) using Lipofectamine 2000 (Thermo Fisher Scientific) according to manufacturer’s protocol. The transfection mixture was added to the medium containing 5% (*w*/*v*) FBS (Gibco, Thermo Fisher Scientific) and devoid of antibiotics. After 4 h, the medium was exchanged for the complete one.

To obtain stably transfected cell lines, HEK293 cells at 75% confluency were transfected with p3xFLAG-CMV-10-CacyBP/SIP or p3xFLAG-CMV-10 (control) plasmid. After 24 h, cells were treated with geneticin (Thermo Fisher Scientific) at a final concentration of 500 ng/mL. The morphology of cells was monitored every day under the light microscope and medium supplemented with geneticin was changed every 2 days. Finally, cells were maintained in medium containing 250 ng/mL geneticin.

### 2.8. Preparation and Propagation of α-Synuclein Seeds in HEK293 Cells

Preparation of α-synuclein seeds was performed according to the method elaborated by the Nieznański group (Nencki Institute of Experimental Biology PAS). Briefly, α-synuclein (1 mg/mL) was incubated for 4 days at 37 °C with 400 rpm agitation (ThermoMixer C, Eppendorf) in a 100 mM acetate buffer containing 1 mM PMSF and 0.02% NaN_3,_ pH 4.7. Then, the sample was centrifuged at 100,000 rpm for 30 min at 4 °C (Sorval MTX 150 Series Micro-Ultracentrifuge, S120-AT3 Fixed Angle Rotor, Thermo Fisher Scientific). After centrifugation, the supernatant was discarded and the pellet containing α-synuclein fibrils was washed twice with PBS containing 0.02% (*w*/*v*) NaN_3_, sonicated, diluted 1:100 in PBS and used for cell transfection.

HEK293 cells stably overexpressing CacyBP/SIP were cultured on glass coverslips previously coated with poly-L-lysine (50 μg/mL) (Sigma-Aldrich) for 24 h. Cells, when confluent (75–80%), were transfected with 1 µg of pcDNA4-3xFLAG plasmid encoding α-synuclein (Table 1). Four hours later, the medium was changed to complete DMEM with 10% (*w*/*v*) FBS supplemented with geneticin (Thermo Fisher Scientific) and cells were cultured for 24 h before a second transfection with α-synuclein seeds. Again, after 4 h, the medium was changed to a complete one and cells were left overnight to form α-synuclein inclusions. Both transfections were performed with the use of Lipofectamine 2000 (Thermo Fisher Scientific) in 5% FBS-containing medium without antibiotics.

### 2.9. Immunofluorescence Staining and Confocal Microscopy

In addition, 24 h after transfection with α-synuclein seeds cells were fixed with 4% (*w*/*v*) paraformaldehyde (Sigma-Aldrich) for 20 min at RT and washed three times for 3 min in PBS. Afterwards, cells were incubated for 10 min in 10 mM PIPES, 25 mM HEPES, 10 mM EGTA, 4 mM MgCl_2_, pH 7.0 (ICCH buffer) containing 50 mM NH_4_Cl and again washed 3 times for 3 min with PBS. The coverslips were then incubated on ice for 4 min in ICCH buffer containing 0.1% Triton X-100. In the subsequent step cells were incubated in PBS containing 5% (*w*/*v*) BSA for 1 h and, then, overnight at 4 °C, with rabbit conformation-specific antibodies against α-synuclein (Abcam) (antibodies that recognize only the misfolded protein) diluted 1:5000 in PBS containing 3% (*w*/*v*) BSA. Then, a secondary anti-rabbit antibody conjugated with Alexa Fluor 488 (Thermo Fisher Scientific), diluted 1:500, in PBS containing 3% (*w*/*v*) BSA were applied at RT for 1.5 h. After final wash with PBS, the coverslips were mounted on slides with media containing DAPI (VectaShield, Sigma-Aldrich). Immunofluorescence was recorded under a confocal microscope (LSM 800, Carl Zeiss, Jena, Germany) equipped with a 63×oil objective in the Laboratory of Imaging Tissue Structure and Function (Nencki Institute of Experimental Biology PAS). The number of α-synuclein inclusions in HEK293 cells was calculated using the ImageJ software (NIH, Bethesda, MD, USA). Only α-synuclein inclusions with total area not smaller than 2 μm^2^ were counted.

### 2.10. Proximity Ligation Assay

In order to visualize α-synuclein-HSP90 or α-synuclein-CacyBP/SIP complexes in HEK293 cells, the proximity ligation assay, PLA (Sigma-Aldric) was applied. This method allows for visualizing proteins which are in close proximity to each other (40 nm or less) i.e., may form a complex. Cells, when confluent (75–80%), were transfected with 1 µg of pcDNA4-3xFLAG plasmid encoding α-synuclein (Table 1). Cells were grown on poly-L-lysine (Sigma-Aldrich) coated glass coverslips and were fixed with 3% (*w*/*v*) paraformaldehyde (Sigma-Aldrich) in buffer containing 60 mM PIPES, 25 mM HEPES, 5 mM EGTA, 4 mM MgCl_2_, pH 7.0 (PHEM buffer) for 20 min at RT. Then, cells were washed with PBS and incubated for 10 min at RT in PHEM buffer containing 50 mM NH_4_Cl. After washing with PBS, cells were permeabilized by 4 min incubation in PHEM buffer containing 0.1% (*w*/*v*) Triton X-100 and washed again with PBS. All the following steps were performed according to the manufacturer’s protocol using reagents (except for primary antibodies) and buffers provided by the manufacturer (Sigma-Aldrich). After blocking, the reaction with primary antibodies, rabbit anti-HSP90α, diluted 1:100 (Abcam), and mouse anti-α-synuclein diluted 1:100 (Abcam), was conducted for 2 h at 37 °C in a humidity chamber. Then, incubation with anti-rabbit PLUS and anti-mouse MINUS PLA-probes diluted 1:5 was carried out for 1 h at 37 °C in a humidity chamber. Following the ligation and amplification steps, the coverslips were immobilized on microscopic slides with a mounting medium containing DAPI. In the control experiment, the ligation step was omitted. Cells were analyzed under a confocal microscope (Zeiss LSM 800, Carl Zeiss), equipped with a 63×oil objective as described above.

### 2.11. Preparation of Protein Lysates from HEK293 Cells

Twenty-four hours after transfection, HEK293 cells were rinsed with ice-cold PBS and harvested in RIPA buffer (Merck Millipore) supplemented with protease inhibitors (Roche, Basel, Switzerland). Cells in RIPA buffer were passed 20 times through a syringe (Micro-Fine^TM^ Plus, BD, Franklin Lakes, NJ, USA) and incubated on ice for 30 min. Protein lysate was centrifuged at 12,000 *g* at 4 °C for 20 min. The supernatant fraction was collected and protein concentration was measured by Bradford’s method (Bio-Rad). Aliquot of the supernatant containing 30 μg of protein was precipitated in ice-cold acetone and kept at −20 °C until use.

### 2.12. Cell Viability Analysis

HEK293 cells with stable overexpression of 3xFLAG-CacyBP/SIP or 3xFLAG alone or non-transfected cells (control) were counted in an automatic cell counter (NanoEnTek, Waltham, MA, USA) and seeded in equal numbers into wells of a 24-well plate. Then, all cells were transfected with plasmid encoding α-synuclein, left for 24 h and transferred to a 96-well plate. After 7 h cells overexpressing 3xFLAG-CacyBP/SIP or 3xFLAG alone were treated with 5 μM rotenone (Sigma-Aldrich) while control cells with an equivalent volume of solvent (96% ethanol) and left for 18 h. Then, the MTS assay (Promega, Madison, WI, USA) was performed to analyze cell viability according to the manufacturer’s protocol. The level of formazan was measured by recording changes in absorbance at 490 nm using a microplate reader (Tecan). Viability of cells overexpressing 3xFLAG-CacyBP/SIP or 3xFLAG was compared to the viability of those treated with solvent alone.

### 2.13. SDS-PAGE and Western Blot

Proteins precipitated in ice-cold acetone were centrifuged at 20,000 *g* for 15 min at 4 °C, mixed with Laemmli’s sample buffer, incubated at 95 °C for 5 min and subjected to SDS-PAGE performed according to Laemmli [30]. For checking protein purity and the crosslinking assay, the gel (15%) was stained with Coomassie brilliant blue R250 (Sigma-Aldrich) while, in the case of checking CacyBP/SIP and α–synuclein overexpression, proteins were separated on 15% gels and transferred onto PVDF membrane (0.45 μm pore size, Immobilon-P, Millipore). Electrotransfer was carried out in the transfer chamber (Mini Protein II, Bio-Rad) filled with pre-chilled SDS-free transfer buffer at a constant current of 250 mA for 1.5 h at 4 °C. During transfer, the buffer was stirred with a magnetic bar. Then, the membrane was incubated in PBS containing 4% paraformaldehyde (Sigma-Aldrich) and 0.01% glutaraldehyde (Sigma-Aldrich) for 30 min in a closed box and then washed 4 times, 10 min each, in TBS-T (50 mM Tris, 200 mM NaCl and 0.05% Tween 20, pH 7.5). The membrane was subsequently incubated in TBS-T containing 5% skim milk for 1 h. After that, the primary rabbit polyclonal anti-CacyBP/SIP antibody (Cell Signaling Technology), diluted 1:1000, or mouse anti-α-synuclein antibody (Abcam) diluted 1:1000 was applied and incubation was carried out overnight at 4 °C. Then, the membrane was washed 3 times for 10 min each in TBS-T and allowed to react for 1 h in RT with secondary antibodies: goat anti-rabbit IgG conjugated to HRP (Jackson ImmunoResearch), diluted 1:10,000, or goat anti-mouse IgG conjugated to HRP (Jackson ImmunoResearch), diluted 1:10,000. The mouse monoclonal anti-β-actin horseradish peroxidase (HRP)-conjugated antibody (Sigma-Aldrich), diluted 1:10,000, was used to monitor protein loading. The PVDF membrane was developed with ECL plus (New England Biolabs, Ipswich, MA, USA) followed by exposure against an X-ray film (Kodak, Rochester, NY, USA). The signal intensity was calculated using the Gene Tools software (Syngene, Frederick, MD, USA), with β-actin as a reference protein, in an Ingenius densitometer (Syngene).

### 2.14. Statistical Analysis

Experiments were performed at least in triplicates and results are presented as means ± SEM. Results of aggregation assays (in vitro and in HEK293 cells), dot-blot and MTS assays were analyzed by Student’s t-test using Microsoft Excel. Results from ELISA were analyzed using one-way ANOVA followed by Tukey’s post-hoc test and GraphPad Prism. The level of statistical significance was set at * *p* ≤ 0.05; ** *p* ≤ 0.01; *** *p* ≤ 0.001.

## 3. Results

### 3.1. Effect of CacyBP/SIP on α-Synuclein Aggregation In Vitro

**Figure 1:**
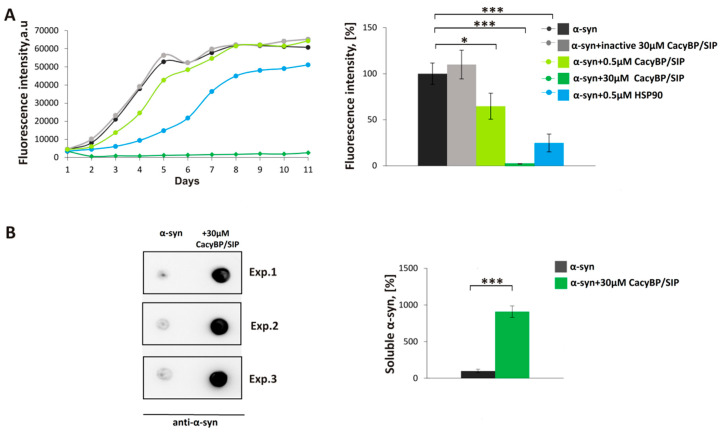
Influence of CacyBP/SIP on α-synuclein aggregation. (**A, left panel**) Representative curves showing ThT fluorescence of 30 µM α-synuclein alone (black) or in the presence of: 30 µM inactive CacyBP/SIP (grey), 0.5 µM CacyBP/SIP (light green), 30 µM CacyBP/SIP (green) or 0.5 µM HSP90 (blue). (**A, right panel**) Statistical analysis of the results for samples taken on day 4 of incubation (*n* = 3). (**B, left panel**) Dot-blots showing α-synuclein immunostaining in the soluble fraction (supernatant) in samples taken on day 4 of α-synuclein aggregation, alone or in the presence of 30 µM CacyBP/SIP and (**B, right panel**) densitometric analysis of the results (*n* = 3). Data, calculated as means ± SEM, are presented as percentage values. * *p* ≤ 0.05, *** *p* ≤ 0.001.

First, we analyzed the influence of CacyBP/SIP on α-synuclein aggregation in vitro by means of thioflavin T (ThT) assay. ThT is a fluorescent dye that increases emission several orders of magnitude after binding to amyloid fibrils [31]. The purity of recombinant proteins used in this experiment and other assays was assessed by SDS-PAGE (Figure S1). Recombinant α-synuclein alone (30 μM) or in the presence of CacyBP/SIP (0.5 or 30 μM) was incubated for 11 days at 37 °C with constant shaking. As it is shown in Figure 1A, 30 μM CacyBP/SIP elicits a drastic decrease in ThT fluorescence. As for 0.5 μM CacyBP/SIP, the decrease is statistically significant on day 4 but not as high as in the case of 0.5 μM HSP90 (positive control). The effect of CacyBP/SIP is specific since no decrease in fluorescence is observed when an inactive protein (heated for 10 min at 96 °C) was used at the same concentration. Remarkably, the effect of 30 μM CacyBP/SIP is evident over the entire course of the experiment.

**Figure 2:**
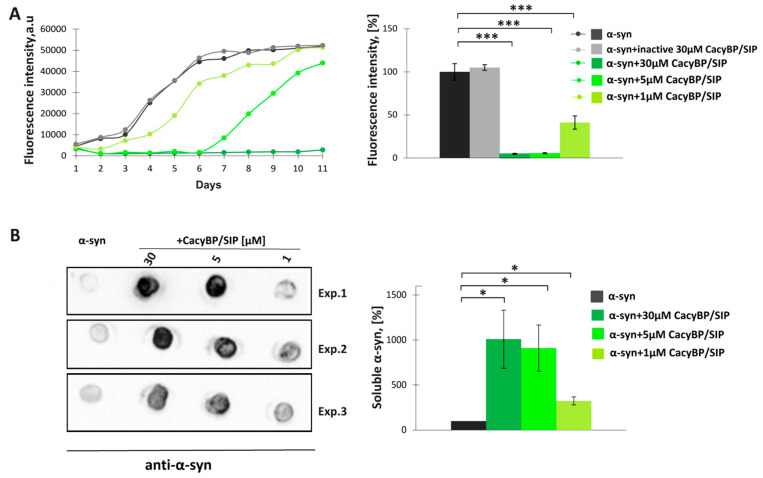
Effect of different concentrations of CacyBP/SIP on α-synuclein aggregation. (**A, left panel**) Representative curves showing ThT fluorescence of 30 µM α-synuclein alone (black) or in the presence of: 30 µM inactive CacyBP/SIP (grey) or 30 µM (dark green), 5 µM (middle green) or 1 µM CacyBP/SIP (light green). (**A, right panel**) Statistical analysis of the results for samples taken on day 4 of incubation (*n* = 3). (**B, left panel**) Dot-blots showing α-synuclein immunostaining in the soluble fraction (supernatant) of samples taken on day 4 of incubation of α-synuclein alone or in the presence of different concentrations of CacyBP/SIP and (**B, right panel**) densitometric analysis of the results (*n* = 3). Data, calculated as means ± SEM, are presented as percentage values. * *p* ≤ 0.05, *** *p* ≤ 0.001.

Subsequently, the amount of soluble α-synuclein on day 4 of incubation was estimated by high-speed ultracentrifugation followed by dot-blot developed with anti-α-synuclein antibody. Analysis of spot intensities shows differences in the amount of soluble α-synuclein between samples containing α-synuclein alone or supplemented with 30 µM CacyBP/SIP (Figure 1B), which is in agreement with ThT results. Since 30 μM CacyBP/SIP is almost totally protected α-synuclein from aggregation and 0.5 μM of CacyBP/SIP had a smaller effect, in the next step, we checked other concentrations of CacyBP/SIP, that is 5 μM and 1 μM, on α-synuclein aggregation. As it can be seen in Figure 2, CacyBP/SIP significantly protected α-synuclein from aggregation when applied at these concentrations.

**Figure 3:**
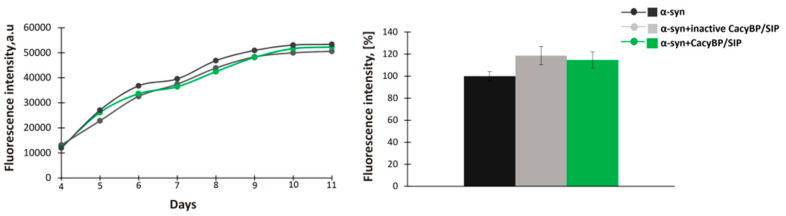
Effect of CacyBP/SIP on later stages of α-synuclein aggregation. (**Left panel**) Representative curves showing ThT fluorescence of 30 µM α-synuclein alone (black) or in the presence of 30 µM: inactive CacyBP/SIP (grey) or CacyBP/SIP (green) added on day 4. (**Right panel**) Statistical analysis of the results of samples taken on day 6 (*n* = 3). Data, calculated as means ± SEM, are presented as percentage values.

A study concerning the HSP70 chaperone has shown that the protein blocks the early stages of aggregation of tau protein, namely nucleation and elongation, and does not influence aggregation when added at later stages [32]. Thus, we tested whether CacyBP/SIP acts in the same fashion. For this purpose, 30 μM α-synuclein was incubated for 4 days at 37 °C and then 30 μM CacyBP/SIP or inactive CacyBP/SIP (negative control) was added. Subsequently, after 2 days, aliquots were taken and ThT fluorescence was measured. As it can be seen in Figure 3, CacyBP/SIP fails to protect α-synuclein from aggregation when added after initiation of the aggregation process.

**Figure 4:**
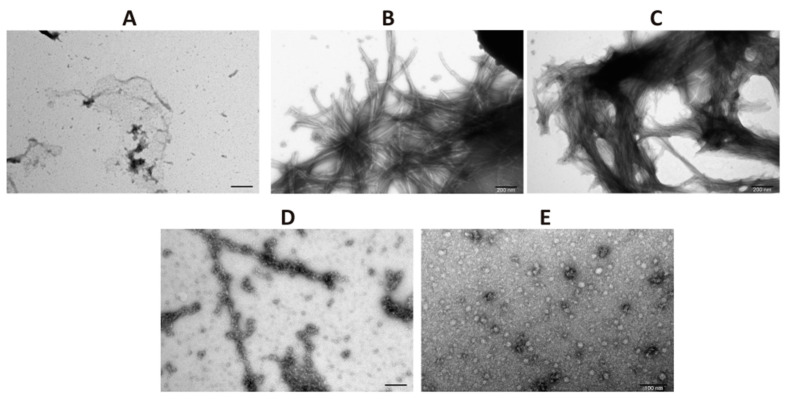
Influence of CacyBP/SIP on α-synuclein aggregation assessed by transmission electron microscopy (TEM). Representative micrographs of α-synuclein obtained before initiation of aggregation (**A**), on day 4 of incubation (**B**), on day 4 of incubation in the presence of: 30 µM inactive CacyBP/SIP (**C**), 15 µM CacyBP/SIP (**D**) or 30 µM CacyBP/SIP (**E**). Scale bar—200 nm (A–D) and 100 nm (**E**).

To confirm the results obtained by ThT assay and ultracentrifugation followed by dot-blot analysis, a transmission electron microscopy (TEM) was applied. For that, on day 4 of aggregation, aliquots were taken and applied to copper grids, followed by negative staining with 2% (*w*/*v*) aqueous solution of uranyl acetate. As it can be seen in Figure 4A, the sample of α-synuclein alone at time point 0 (before initiation of aggregation) does not contain any evident structures that resemble fibers while large amorphous fibers are clearly discernible on day 4 of aggregation (Figure 4B). As expected, CacyBP/SIP inhibits the formation of α-synuclein fibers in a concentration dependent way (Figure 4D,E). TEM images show that, in the presence of CacyBP/SIP, oligomer-like α-synuclein structures are formed. Importantly, these structures represent only a small fraction of α-synuclein species present in the sample. As it was expected, in the presence of inactive CacyBP/SIP, large amorphous fibers of α-synuclein were found (Figure 4C; negative control). Thus, the obtained results are in agreement with those of ThT assay and high-speed centrifugation followed by dot-blot, and show that CacyBP/SIP protects α-synuclein from aggregation when added prior to initiation of this process.

### 3.2. Effect of CacyBP/SIP Domains on α-Synuclein Aggregation In Vitro

**Figure 5:**
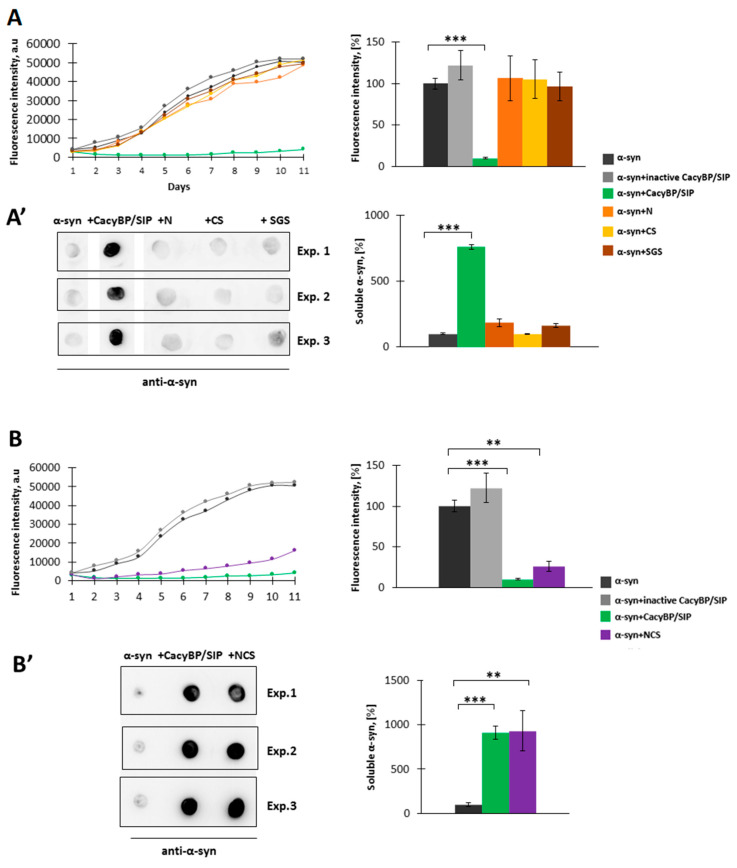
Influence of CacyBP/SIP domains on α-synuclein aggregation. (**A, left panel**) Representative curves showing ThT fluorescence of 30 µM α-synuclein alone (black) or in the presence of 30 µM: inactive CacyBP/SIP (grey), CacyBP/SIP (green), N domain (orange), CS domain (yellow) or SGS domain (brown). (**A, right panel**) Statistical analysis of the results (*n* = 3). (**A’, left panel**) Dot-blots showing α-synuclein immunostaining in the soluble fraction and (**A’, right panel**) statistical analysis of the results (*n* = 3). **(B, left panel)** Representative curves showing ThT fluorescence of 30 µM α-synuclein alone (black) or in the presence of 30 µM: inactive CacyBP/SIP (grey), CacyBP/SIP (green) or NCS fragment of CacyBP/SIP (violet) (**B, right panel**) Statistical analysis of the results (*n* = 3). (**B’, left panel**) Dot-blots showing α-synuclein immunostaining in the soluble fraction and (**B’, right panel**) statistical analysis of the results (*n* = 3). In all cases, samples were taken on day 4 of incubation and data, calculated as means ± SEM, are presented as percentage values. ** *p* ≤ 0.01, *** *p* ≤ 0.001.

CacyBP/SIP is composed of three major domains: The N-terminal (N, residues 1–77), middle (CS, residues 74–178) and the C-terminal (SGS, residues 178–229) [27]. Hence, in the next step, we attempted to identify domain(s) of this protein responsible for inhibition of α-synuclein aggregation in vitro. We performed ThT assay and high-speed centrifugation followed by dot-blot analysis as previously described. The obtained results show that none of the CacyBP/SIP domains protects α-synuclein from aggregation (Figure 5A,A’).

We also examined whether CacyBP/SIP fragment overlapping the N-terminal (N) and middle (CS) domains, called NCS (residues 1–178), is effective in α-synuclein aggregation. According to results presented in Figure 5B,B’, this fragment inhibits α-synuclein aggregation to a similar extent as full-length CacyBP/SIP.

### 3.3. Direct Interaction between α-Synuclein and CacyBP/SIP

**Figure 6:**
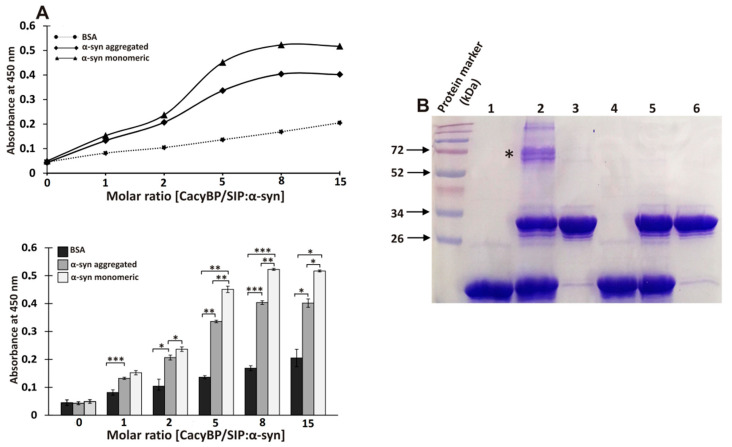
Interaction between α-synuclein and CacyBP/SIP studied by ELISA (**A**) and chemical crosslinking (**B**). (**A**) Upper panel shows absorbance measured at different molar ratio of CacyBP/SIP (concentration 0, 3.89, 7.78, 19.45, 31.12, and 58.35 µM) to α-synuclein (3.89 µM) while the lower one shows quantitative analysis of the results obtained from 3 independent experiments. Data are presented as means ± standard errors (SEM); * *p* ≤ 0.05, ** *p* ≤ 0.01, *** *p* ≤ 0.001. (**B**) 15% polyacrylamide gel stained with Coomassie brilliant blue R250. α-synuclein (30 μM) was mixed with CacyBP/SIP (30 μM) Lane 1 and 4, α-synuclein alone; Lanes 2 and 5, mixture of α-synuclein and CacyBP/SIP; Lanes 3 and 6, CacyBP/SIP alone. Proteins were incubated with (lanes 1–3) and without crosslinking agent (lanes 4–6) and then 15 µl of reaction mixture was applied on the gel. “*” indicates the α-synuclein-CacyBP/SIP crosslinking product. A representative experiment, out of 3 performed, is shown.

Interaction between α-synuclein and CacyBP/SIP was tested by ELISA and a crosslinking experiment. As to ELISA, monomeric or aggregated α-synuclein (incubated 4 days in conditions described for ThT assay) or BSA (as a negative control) was coated on wells and then CacyBP/SIP was applied. The obtained results show higher value of absorbance for α-synuclein than for BSA (control) which suggests that α-synuclein interacts with CacyBP/SIP (Figure 6A). Notably, the results suggest that monomeric α-synuclein binds more strongly to CacyBP/SIP than the aggregated one. In order to confirm these results, chemical crosslinking of monomeric α-synuclein with CacyBP/SIP was performed. Figure 6B (lane 2, asterisk) shows the presence of a band corresponding to the crosslinking product formed between α-synuclein and CacyBP/SIP.

**Figure 7:**
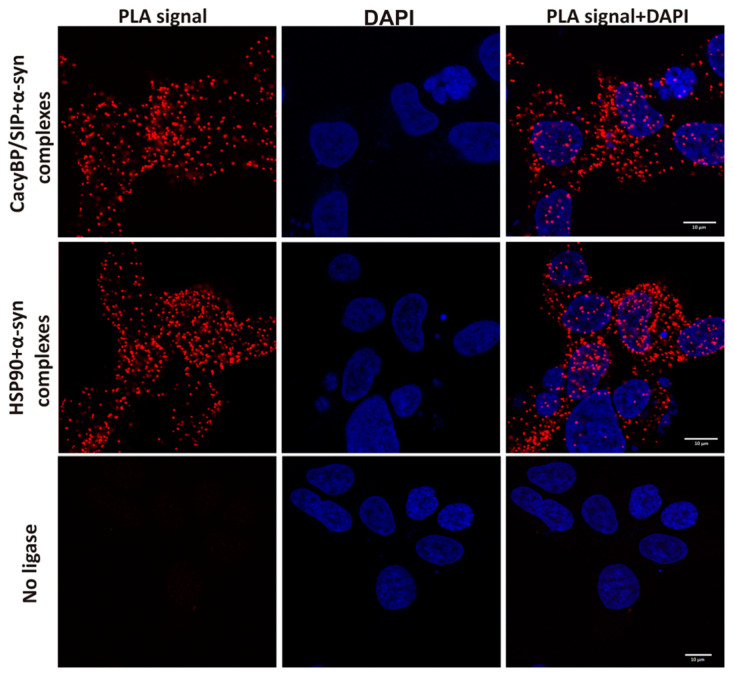
Presence of complexes formed between α-synuclein and CacyBP/SIP or HSP90 in HEK293 cells visualized by PLA (representative images). Complexes of examined proteins are shown as red dots; cell nuclei, stained with DAPI, are in blue. HSP90 was used as a positive control. Scale bar–10 μm.

To check whether α-synuclein interacts with CacyBP/SIP in the cell, we applied the PLA assay with the use of HEK293 cells. This method allows visualizing complexes formed between proteins in close proximity to each other (40 nm or less). As it can be seen in Figure 7, red dots that represent complexes formed between α-synuclein and CacyBP/SIP or α-synuclein and HSP90 (positive control) are clearly visible throughout the cytoplasm. As expected, when ligase was omitted, no such red dots were visible (negative control). Altogether, these results show that the α-synuclein-CacyBP/SIP interaction is direct.

### 3.4. Influence of CacyBP/SIP on α-Synuclein Aggregation in HEK293 Cells and Their Viability

**Figure 8:**
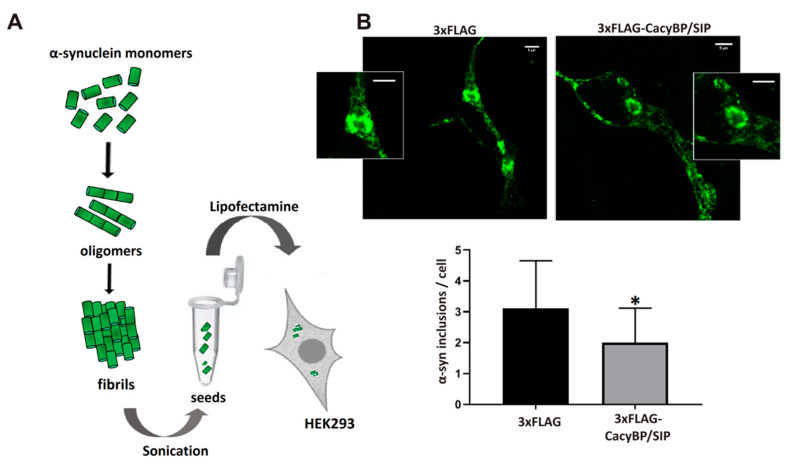
Influence of CacyBP/SIP on the number of α-synuclein aggregates in HEK293 cells. (**A**) A scheme showing preparation of α-synuclein seeds and their delivery to HEK293 cells. (**B, upper part**) Representative immunofluorescence staining performed with the use of primary conformation-specific anti-α-synuclein antibody. Aggregates/inclusions of α–synuclein are visible in green. Insert shows enlargement of inclusion. (**B, lower part**) Statistical analysis of the results from 3 independent experiments (30 inclusion-containing cells were analyzed) are presented as means ± standard errors (SEM); * *p* ≤ 0.05. Scale bar—5 μm.

In order to check whether CacyBP/SIP protects α-synuclein from aggregation in the cell, we established the HEK293 cell line with stable overexpression of 3xFLAG-CacyBP/SIP (Figure S2A). These cells were then transfected with plasmid encoding α-synuclein (Figure S2B) and with α-synuclein seeds. Scheme showing preparation of α-synuclein seeds and their delivery to HEK293 cells is shown in Figure 8A. The resulting α-synuclein inclusions in HEK293 cells were stained with conformation-specific monoclonal anti-α-synuclein antibody and analyzed under the confocal microscope. The obtained results demonstrate that less α-synuclein inclusions have been formed in cells overexpressing 3xFLAG-CacyBP/SIP (Figure 8B) than in control cells. For control experiments, CacyBP/SIP overexpressing cells were transfected only with seeds (Figure S3A) or only with plasmid encoding α-synuclein (Figure S3B).

**Figure 9:**
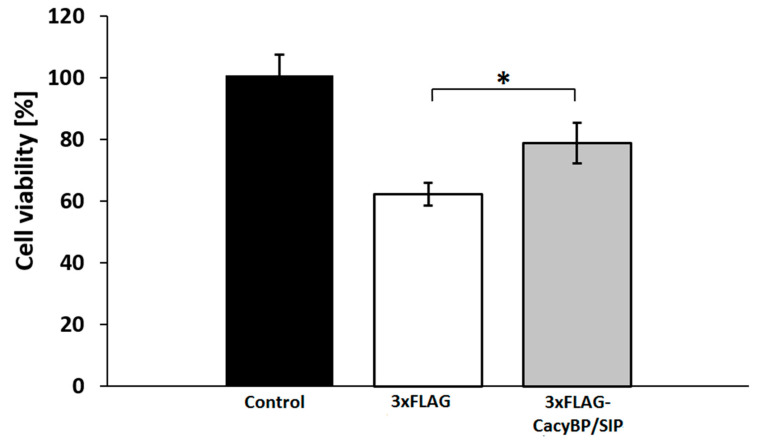
Viability of HEK293 cells overexpressing CacyBP/SIP after rotenone treatment. Cells treated with solvent (black bar), cells transfected with 3xFLAG (white bar) or with 3xFLAG-CacyBP/SIP (grey bar) treated with rotenone. Cell viability was quantified using an MTS assay. Data obtained from 3 independent experiments, calculated as means ± SEM, are presented as percentage values. * *p* ≤ 0.05.

To understand the possible role of CacyBP/SIP in PD pathology, we measured viability of HEK293 cells overexpressing 3xFLAG-CacyBP/SIP and control ones (expressing FLAG tag alone) after rotenone treatment. As it can be seen in Figure 9 an increased CacyBP/SIP level augments viability of these cells by about 16%.

## 4. Discussion

Initially, α-synuclein was identified as a major component of LBs, that is, inclusions mostly found in dopaminergic neurons of substantia nigra of PD brain. LBs are a major hallmark of PD and some other neurodegenerative diseases, collectively called synucleinopathies [33]. Chaperones and co-chaperones are essential players in the regulation of cellular proteostasis; in particular, they protect cells from pathogenic aggregation of misfolded proteins [34]. For instance, it has been shown that, in vitro, HSP70 alone or in cooperation with different chaperones/co-chaperones inhibits formation of α-synuclein fibrils [35,36] and tau aggregates [32], and reduces polyglutamine (polyQ)-induced toxicity when exogenously added to cultured cells [37]. Other chaperones such as HSP60 and HSP40 have been shown to suppress α-synuclein fibrillization in vitro and to block the polyQ-induced toxicity, respectively [38,39]. In turn, overexpression of αB-crystallin and HSP27, members of small heat shock protein family (sHSP), significantly reduces the intracellular aggregation of α-synuclein and inhibits cytotoxicity of α-synuclein fibrils [40].

In the present work, we have analyzed the influence of a novel HSP90 co-chaperone, CacyBP/SIP, on α-synuclein aggregation. We have found that CacyBP/SIP protects α-synuclein from aggregation. Moreover, we have identified the fragment of CacyBP/SIP crucial for preventing α-synuclein aggregation. These results are in agreement with the recent work showing influence of CacyBP/SIP on aggregation of other proteins e.g., citrate synthase [20]. The results of ThT analysis obtained for full length CacyBP/SIP were verified by ultracentrifugation followed by dot-blot and by transmission electron microscopy (TEM). As to TEM, in the presence of CacyBP/SIP, a small amount of structures that may represent α-synuclein oligomers was found. Similar results, i.e., small oligomers of α-synuclein were observed when HSP90 was present during aggregation of α-synuclein [17]. Moreover, it has been shown that oligomers formed in the presence of HSP90 are not toxic for cells. To better understand the nature and function of α-synuclein oligomers formed in the presence of CacyBP/SIP, more studies are required. In this work, we have also analyzed the refolding activity of CacyBP/SIP towards α-synuclein and found that CacyBP/SIP protects α-synuclein from aggregation only when added during the initial phase of the process. This result is in agreement with data obtained by ELISA, which suggest higher affinity of CacyBP/SIP for monomeric than for the aggregated form of α-synuclein. In view of this result, it would be important to establish Kd and stoichiometry of the interaction between CacyBP/SIP and monomeric or fibrillized α-synuclein. Of note, ELISA and crosslinking assays point to a direct interaction between CacyBP/SIP and α-synuclein. However, applying another method, for instance, Forster resonance energy transfer (FRET), could additionally confirm direct interaction between these two proteins.

Importantly, we have found that, in HEK293 cells, α-synuclein forms complexes with CacyBP/SIP and detected slightly less α-synuclein inclusions in cells overexpressing CacyBP/SIP than in control ones. In addition, we have checked whether CacyBP/SIP overexpressing HEK293 cells treated with rotenone are more viable than control ones. We used this agent since it evokes behavioral and histopathological symptoms of PD [41]. The obtained results showed a protective effect of CacyBP/SIP on the viability of HEK293 cells which is in agreement with previously reported data obtained for CacyBP/SIP overexpressing cells treated with other stress factors [21].

Altogether, in this work, we demonstrate for the first time that CacyBP/SIP is able to protect α-synuclein from aggregation in vitro and in the cellular model. A question arises whether CacyBP/SIP plays a universal role in maintaining cellular proteostasis or whether it discriminates its cellular targets for folding. To dispel these doubts, additional studies should be performed. Anyway, the presented research represents a new concept of dealing with α-synuclein aggregation/toxicity. Continuation of this work may point to CacyBP/SIP as a potent factor able to attenuate α-synuclein pathology and may create basis for development of new therapeutic strategies applicable in treatment of PD and other synucleinopathies.

## Figures and Tables

**Table 1:** List of plasmids used for protein purification and for cell transfection.

Plasmid	Description	Source/Reference
pET28a	plasmid for protein expression in *E. coli* under the T7 promoter encoding protein with 6xHis tag	Novagene
pET28a-CacyBP/SIP	plasmid encoding CacyBP/SIP with 6xHis tag	[27]
pET28a-CacyBP/SIP 1–77	plasmid encoding N-terminal (N) domain (residues 1–77) of CacyBP/SIP with 6xHis tag	[27]
pET28a-CacyBP/SIP 74–178	plasmid encoding middle (CS) domain (residues 74–178) of CacyBP/SIP with 6xHis tag	[27]
pET28a-CacyBP/SIP 178–229	plasmid encoding C-terminal (SGS) domain (residues 178–229) of CacyBP/SIP with 6xHis tag	[27]
pET28a-CacyBP/SIP 1–178	plasmid encoding fragment of CacyBP/SIP covering N-terminal (N) and CS domains (NCS; residues 1–178) of CacyBP/SIP with 6xHis tag	[27]
pET28a-α-synuclein	plasmid encoding α-synuclein with 6xHis tag	present work
p3xFLAG-CMV-10	plasmid for protein expression in eukaryotic cells encoding 3xFLAG tag	Sigma-Aldrich
p3xFLAG-CMV-10 -CacyBP/SIP	plasmid for protein expression in eukaryotic cells encoding 3xFLAG-CacyBP/SIP	[20]
pcDNA4-α-synuclein-3xFLAG	plasmid for protein expression in eukaryotic cells encoding α-synuclein-3xFLAG	U. Dettmer, Harvard Medical School, Boston, USA

## Supplementary Materials

The following are available online at https://www.mdpi.com/2073-4409/9/10/2254/s1, Figure S1. Coomassie stained 15% SDS gel showing the purity of recombinant α-synuclein, CacyBP/SIP and its domains: N, CS, SGS and the NCS fragment. Figure S2. Overexpression of 3xFLAG-CacyBP/SIP and of α-synuclein-3xFLAG in HEK293 cells. Figure S3. Immunofluorescence staining of HEK293 cells with conformation-specific anti-α-synuclein antibody.

## Abbreviations

BSA: bovine serum albumin; CacyBP/SIP, Calcyclin Binding Protein/Siah-1 Interacting Protein; DTT, dithiothreitol; EGTA, ethylene glycol-bis(α-aminoethyl ether)-N,N,N’,N’-tetraacetic acid; HRP, horseradish peroxidase; HSP, heat shock protein; sHSP, small heat shock protein; IPTG, isopropyl α-D-1-thiogalactopyranoside; MTS assay, assay to measure cell viability; PBS, phosphate buffered saline; PBS-T, PBS containing 0.05% Tween 20; PMSF, phenylmethylsulfonyl fluoride, SDS, sodium dodecyl sulfate; TBS, tris-buffered saline; TBS-T, tris-buffered saline containing 0.05% Tween 20; TEM, transmission electron microscopy; ThT, thioflavin T.
