# Supplementary material for: HSP90 Co-Chaperone, CacyBP/SIP, Protects α-Synuclein from Aggregation
Source: Cells. 2020 Oct 8;9(10):2254. doi: 10.3390/cells9102254 (PMC7600563; doi:10.3390/cells9102254)
Supplement: Supplementary file 1 [file cells-09-02254-s001.pdf]

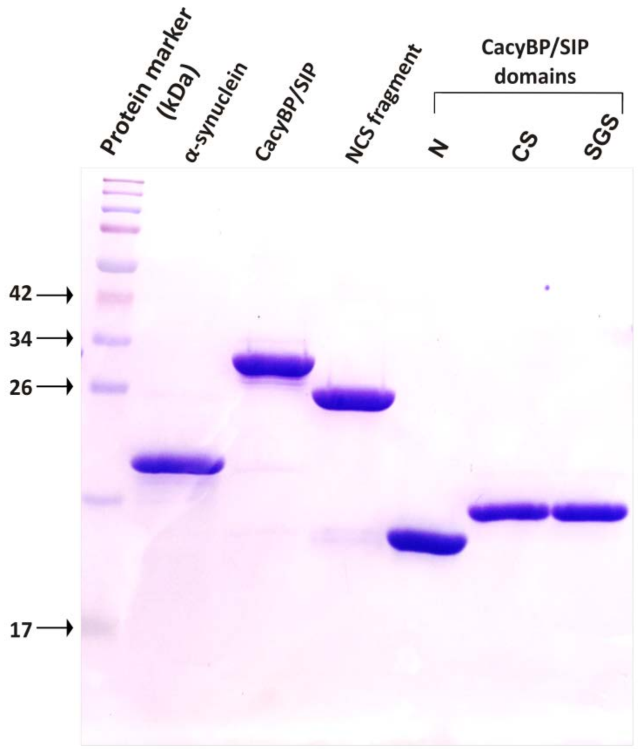

Figure 1. Coomassie stained 15% SDS gel showing the purity of recombinant α-synuclein, CacyBP/SIP and its domains: N, CS, SGS and the NCS fragment.

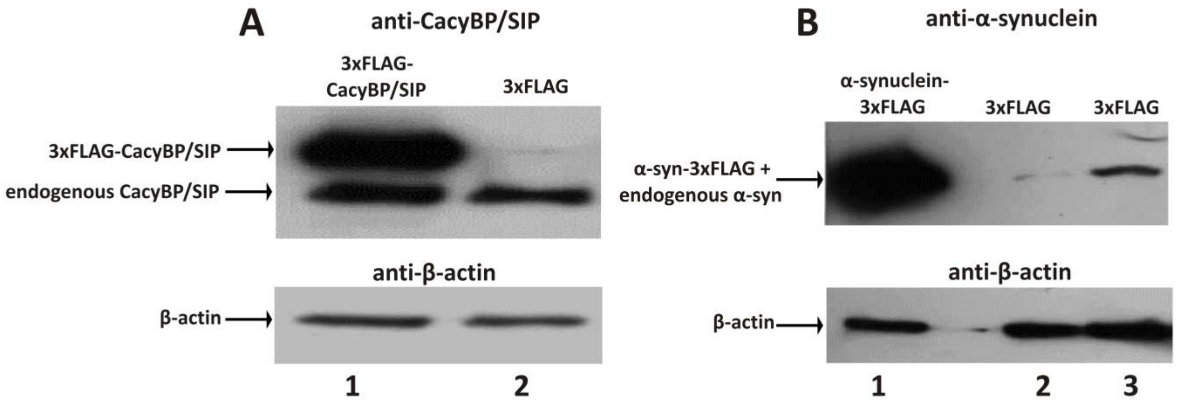

Figure 2. Overexpression of 3xFLAG-CacyBP/SIP and of α-synuclein-3xFLAG in HEK293 cells. 15% SDS-PAGE followed by Western blot developed with anti-CacyBP/SIP **(A)** and anti-α-synuclein **(B)** antibodies, respectively. **(A)** Lane 1- 30 µg of protein from lysate of cells transfected with p3xFLAG-CacyBP/SIP and lane 2 - 30 µg of protein from lysate of cells transfected with p3xFLAG (control). **(B)** Lane 1- 30 µg of protein from lysate of cells transfected with pcDNA4-α-synuclein-3xFLAG, lane 2 and 3 - 30 µg and 50 µg of protein from lysate of cells transfected with p3xFLAG (control), respectively. Of note, CacyBP/SIP and 3xFLAG CacyBP/SIP are visible as two separate bands, while α-synuclein endogenous and overexpressed with FLAG tag, due to low molecular weight of α-sunuclein is not separated in 15% SDS gel.

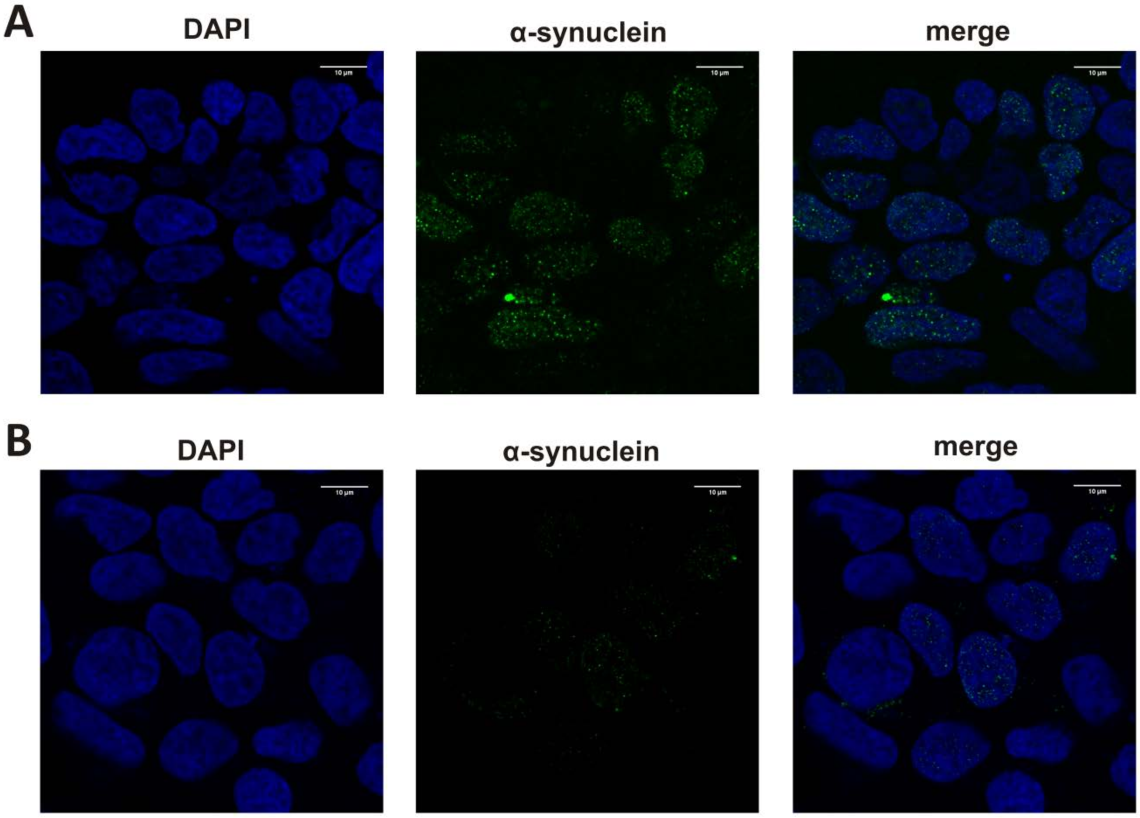

Figure S3. Immunofluorescence staining of HEK293 cells with conformation-specific anti-α-synuclein antibody. (A) Cells transfected with seeds and not with plasmid encoding α-synuclein**. (B)** Cells transfected with plasmid encoding α-synuclein and not with seeds. α-synuclein – green, nuclei, stained with DAPI – blue. Scale bar - 10 µm.
